# Differentiation between benign and malignant orbital tumors using deep transfer learning features and hand-crafted radiomics features from traditional CT imaging

**DOI:** 10.3389/fonc.2026.1641433

**Published:** 2026-04-22

**Authors:** Weitao Huang, Xingjian Xu, Xiaowei Han, Guozheng Zhang

**Affiliations:** 1 Department of Radiology, The Quzhou Affiliated Hospital of Wenzhou Medical University, Quzhou People’s Hospital, Quzhou, China; 2 Zhejiang Chinese Medical University, Hangzhou, China

**Keywords:** CT, deep learning, differentiation diagnosis, orbital lesions, radiomics

## Abstract

**Objective:**

To evaluate the diagnostic performance of deep learning−based radiomics (DL) and hand−crafted radiomics (HCR) in differentiating benign from malignant orbital tumors.

**Methods:**

A retrospective analysis was performed on CT data from 145 patients (48 benign, 97 malignant) diagnosed between December 2014 and March 2024. Two radiologists independently assessed conventional CT semantic features (e.g., lesion location, margin definition, internal density homogeneity, calcification, necrosis, and enhancement pattern). Deep transfer learning (DTL) extracted DL features, while traditional methods were used to obtain HCR features. Feature fusion, selection, and modeling were performed using the Least Absolute Shrinkage and Selection Operator (LASSO). Pathological diagnosis served as the gold standard. Model performance was evaluated using receiver operating characteristic (ROC) curves. A nomogram integrating clinical data and significant semantic features was constructed for visualization. The DeLong test and decision curve analysis (DCA) assessed model effectiveness.

**Results:**

Multivariate analysis confirmed that homogeneous enhancement and ill−defined/infiltrative margins were independent CT features differentiating benign from malignant tumors. A total of 14 HCR and 30 DL features were extracted; 36 features were retained after fusion. The HCR, DL, fused, and nomogram models achieved AUCs of (0.859/0.816), (0.957/0.826), (0.986/0.811), and (0.975/0.837) in the training and test cohorts, respectively. The DeLong test showed no significant difference between the fused model and the nomogram in either cohort (*P* = 0.090 and P = 0.198), whereas differences for other model pairs were significant (*P* < 0.05). DCA indicated that the nomogram provided higher clinical utility.

**Conclusion:**

The fused model outperformed single radiomics approaches in accuracy. The nomogram, which integrates clinical data and semantic features, demonstrated superior predictive performance and may support clinical decision−making, particularly for patients who cannot undergo invasive procedures.

## 1 Introduction

Orbital diseases encompass a wide range, from benign lesions to malignant tumors, each potentially leading to significant morbidity and functional impairment. The orbit is a complex anatomical structure that includes the eyeball and its associated tissues, such as the eyelids, conjunctiva, lacrimal apparatus, extraocular muscles, and the orbital walls. Due to the diversity of tissues involved, the types of potential lesions are complex and highly varied (1). Benign lesions typically require only observation or medical treatment, while malignant lesions usually necessitate surgical intervention (2). Therefore, distinguishing between benign and malignant lesions is clinically significant. However, due to the specificity of clinical presentation and biological characteristics, diagnosing orbital lesions remains a challenging task (3, 4).

With the advancement of medical imaging technologies, such as ultrasound, computed tomography (CT), and magnetic resonance imaging (MRI), valuable information can be obtained to aid in the diagnosis of orbital diseases. MRI, in particular, provides accurate anatomical descriptions, allowing for differentiation between benign and malignant lesions and providing qualitative diagnoses of orbital diseases (5, 6). Despite the advantages of medical imaging, histopathological examination remains the gold standard for diagnosing orbital diseases. However, obtaining tissue samples often requires invasive procedures, which carry inherent risks, including surgical complications and biopsy-related side effects such as functional impairment, disfigurement, and vision loss (7).

Therefore, there is an urgent need to develop new non-invasive diagnostic tools to differentiate orbital lesions. One promising approach is to combine deep learning with radiomics in CT imaging. Radiomics extracts quantitative features from medical images and further analyzes image characteristics that cannot be observed by the human eye using advanced algorithmic models (8, 9). Radiomics has shown tremendous potential in cancer diagnosis and treatment evaluation (10, 11). As a branch of artificial intelligence, deep learning has demonstrated significant accuracy in analyzing complex imaging data, primarily through the use of filter matrices and convolutional neural networks (CNN) to extract image features. This requires large labeled datasets to understand the underlying relationships in the data (12). Deep transfer learning (DTL) is a process that employs a pre-trained deep learning network (13) and fine-tunes it to learn new tasks, enabling deep learning radiomics to be applied to small datasets. This strategy has become a research hotspot in recent years (14–16). These findings make it an ideal tool for distinguishing benign from malignant orbital tumors.

This study aims to explore the potential of deep learning-based CT radiomics in differentiating benign and malignant orbital tumors and to develop and validate a deep learning radiomics nomogram (DLRN) for the differential diagnosis of orbital benign and malignant tumors, providing a non-invasive, accurate, and reliable alternative to traditional diagnostic methods. To this end, we developed and validated a CT−based diagnostic framework that integrates deep transfer learning features, hand−crafted radiomics features, and significant conventional CT semantic features. Compared with previous orbital imaging studies, our work focuses on routine contrast−enhanced CT and further provides a clinically interpretable nomogram, which may offer a practical non−invasive tool for preoperative assessment.

## 2 Materials and methods

### 2.1 Patients

**Figure 1:**
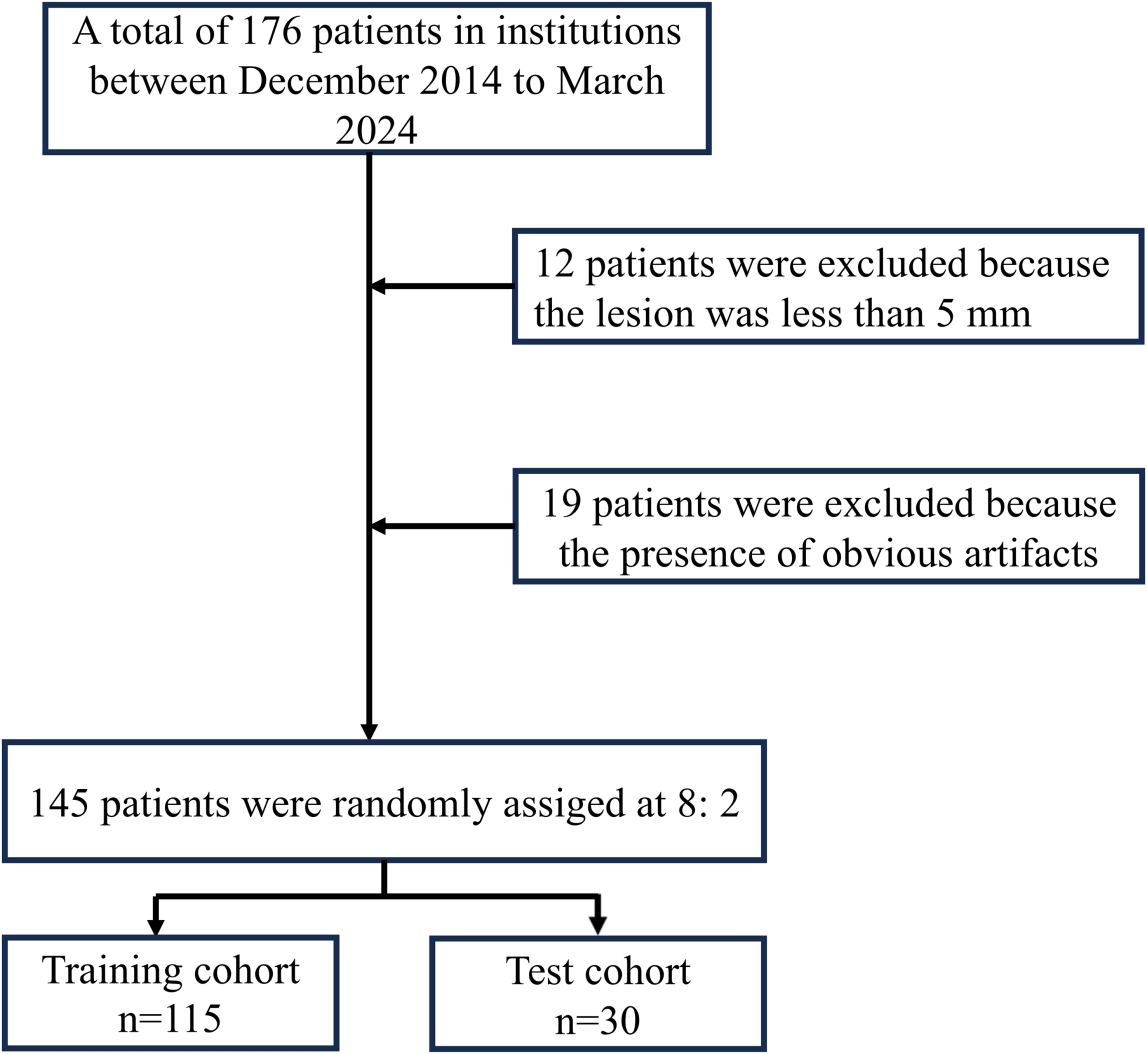
Flow chart of study inclusion.

**Figure 2:**
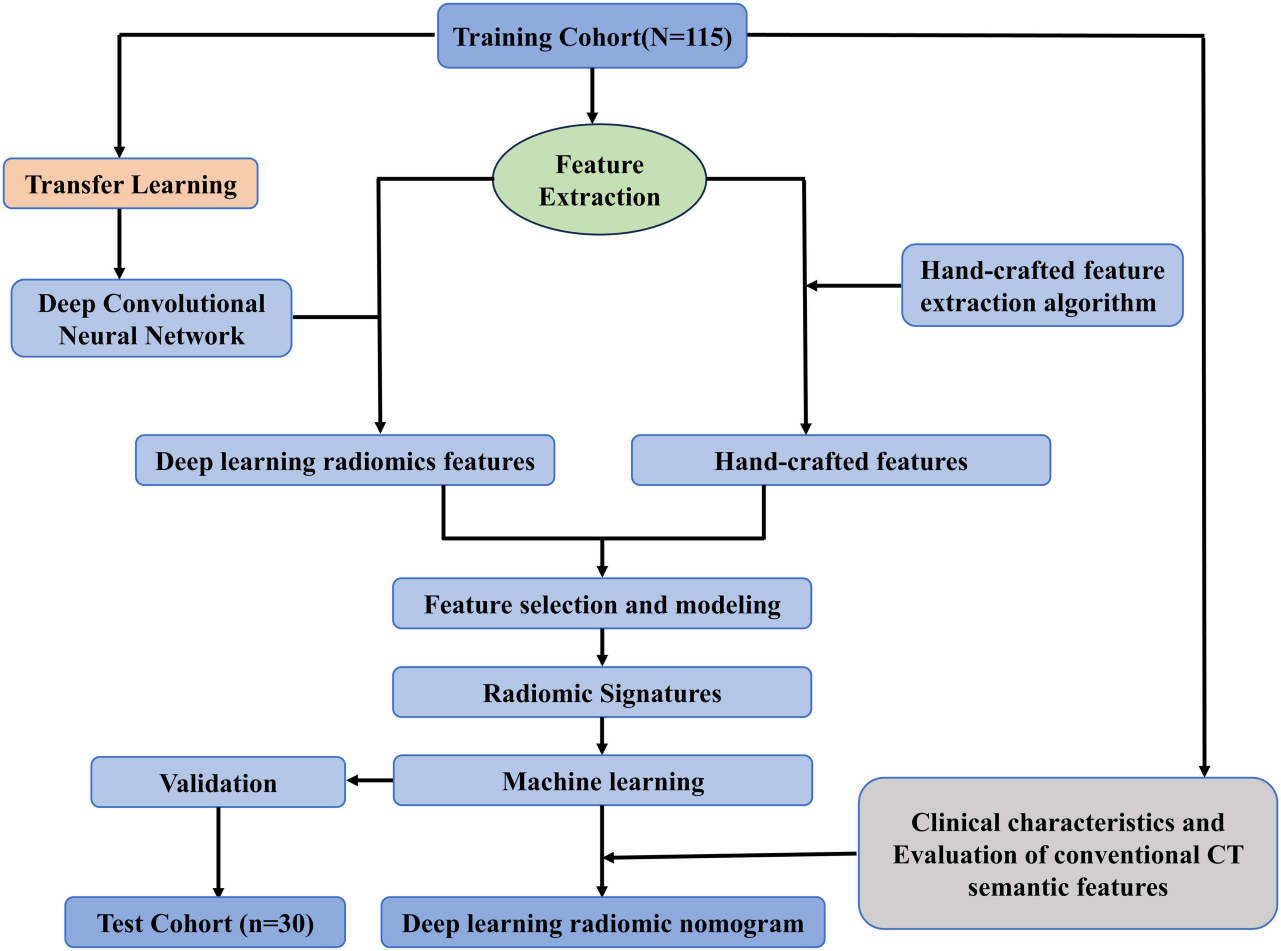
Study flowchart.

**Figure 3:**
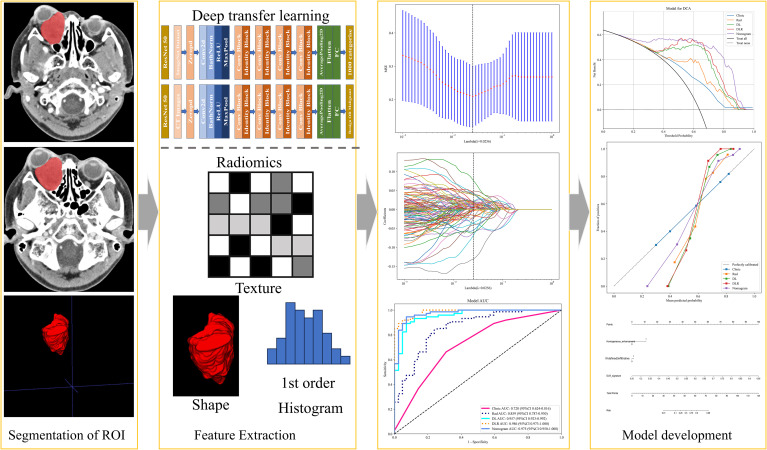
Deep learning radiomics workflow.

Approval for this study was obtained from the Medical Ethics Committee of Quzhou People’s Hospital (Approval NO: 2024-139). We retrospectively analyzed the contrast-enhanced CT imaging data of 176 patients with orbital diseases diagnosed and treated at our hospital between December 2014 and March 2024. Data for this retrospective cohort study were accessed from the hospital’s electronic health records system on May 10, 2024.The inclusion criteria were as follows: (1) patients with malignant and benign tumors confirmed by postoperative pathology; (2) CT examination within 2 weeks prior to surgery; (3) complete clinical and pathological data. The exclusion criteria were as follows: (1) tumor size < 5 mm; (2) motion or susceptibility artifacts that impaired correct segmentation. Ultimately, 97 patients with malignant tumors and 48 patients with benign tumors were enrolled in the study. Details of patient selection are shown in **Figures 1**–**3** display the study flowchart and the deep transfer learning workflow, which include steps such as case collection and grouping, image preprocessing, feature extraction, feature analysis, and model construction. All patients were randomly assigned to the training group and the validation group at an 8:2 ratio.

### 2.2 Clinical data and CT image acquisition

The age and gender data of all patients were collected from the clinical medical records system. All CT images were acquired using a 64-slice spiral CT scanner. The scanning parameters were as follows: tube voltage of 120 kV, automatic tube current modulation, with a set noise index. All images (slice thickness: 1.25 mm) were reconstructed using soft tissue reconstruction (window width: 100, window level: 45) and were then processed and analyzed based on the obtained soft tissue window images.

### 2.3 Evaluation of conventional CT semantic features

Conventional CT semantic features, which are well-established diagnostic indicators, were evaluated for each tumor. Two attending radiologists specializing in head and neck imaging (with 5 and 10 years of experience, respectively), blinded to all clinical and pathological outcomes, independently reviewed the soft-tissue window images. The assessed features included: (1) Lesion Location (intraconal, extraconal, or trans-spatial); (2) Margin status (well-defined or ill-defined/infiltrative); (3) Internal Density Homogeneity (homogeneous or heterogeneous); (4) Internal Characteristics (presence of calcification and necrosis/cystic change); and (5) Enhancement Pattern (homogeneous, or heterogeneous including rim enhancement). Discrepancies were resolved by consensus with a senior radiologist. All qualitative assessments were then quantified into categorical variables (e.g., Margin: well-defined=0, ill-defined=1) for statistical analysis and model integration.

### 2.4 Image segmentation

Precise segmentation of orbital lesions is essential for image analysis. A radiologist (M.Q.) used open-source ITK-SNAP software (version 3.6.0; http://www.itk-snap.org) to perform manual segmentation. The three-dimensional volume of interest (ROI) was delineated by stacking region-of-interest slices to cover the entire tumor. To verify interobserver reproducibility, 30 randomly selected lesions were segmented by another radiologist. The intraclass correlation coefficient (ICC) was used to determine inter-observer agreement on radiomics features, with ICC ≥ 0.8 considered acceptable reliability.

### 2.5 HCR feature extraction

To prevent data leakage, feature selection was performed using only the training group data. Before feature extraction, all images were standardized. All images underwent isotropic interpolation, generating isotropic 3D data with a pixel spacing of 1 mm, and these were standardized as the input images for grayscale feature extraction and filtering transformations. Feature extraction algorithms followed the Image Biomarker Standardization Initiative. Hand-crafted radiomics (HCR) features were extracted using the open-source Python package Pyradiomics (http://pypi.org/project/pyradiomics/). HCR features included first-order features, shape, Gray Level Co-occurrence Matrix (GLCM), Gray Level Size Zone Matrix (GLSZM), Gray Level Run Length Matrix (GLRLM), Neighborhood Gray Tone Difference Matrix (NGTDM), and Gray Level Dependence Matrix (GLDM).

### 2.6 DL feature extraction

**Figure 4:**
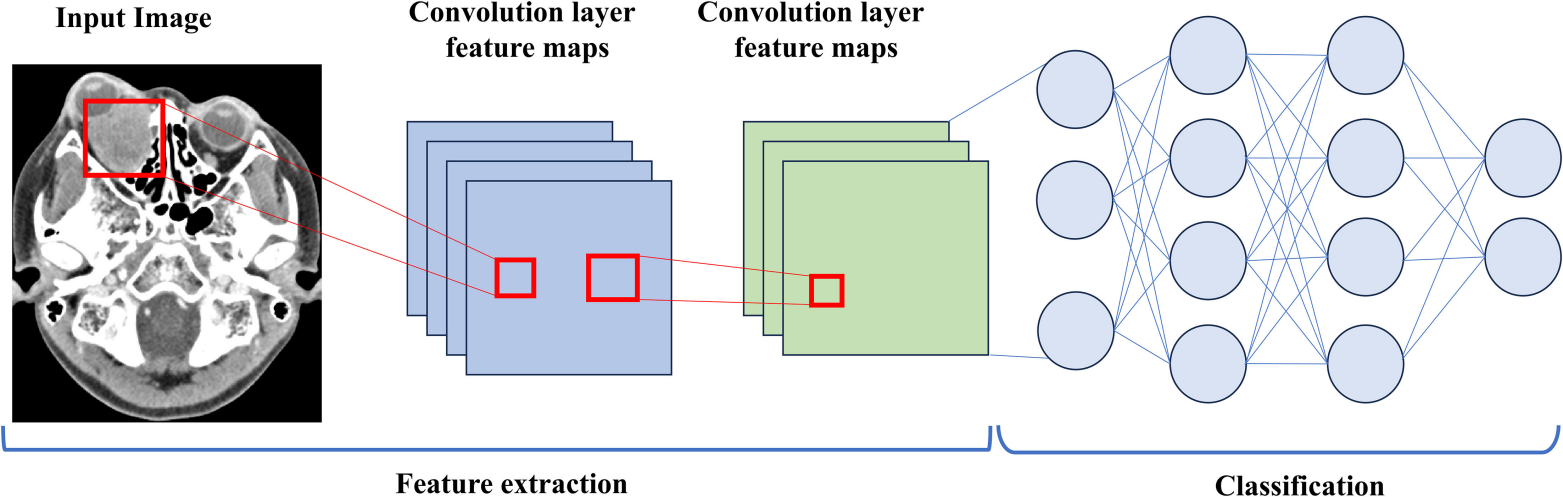
A simple convolutional neural network architecture.

**Figure 5:**
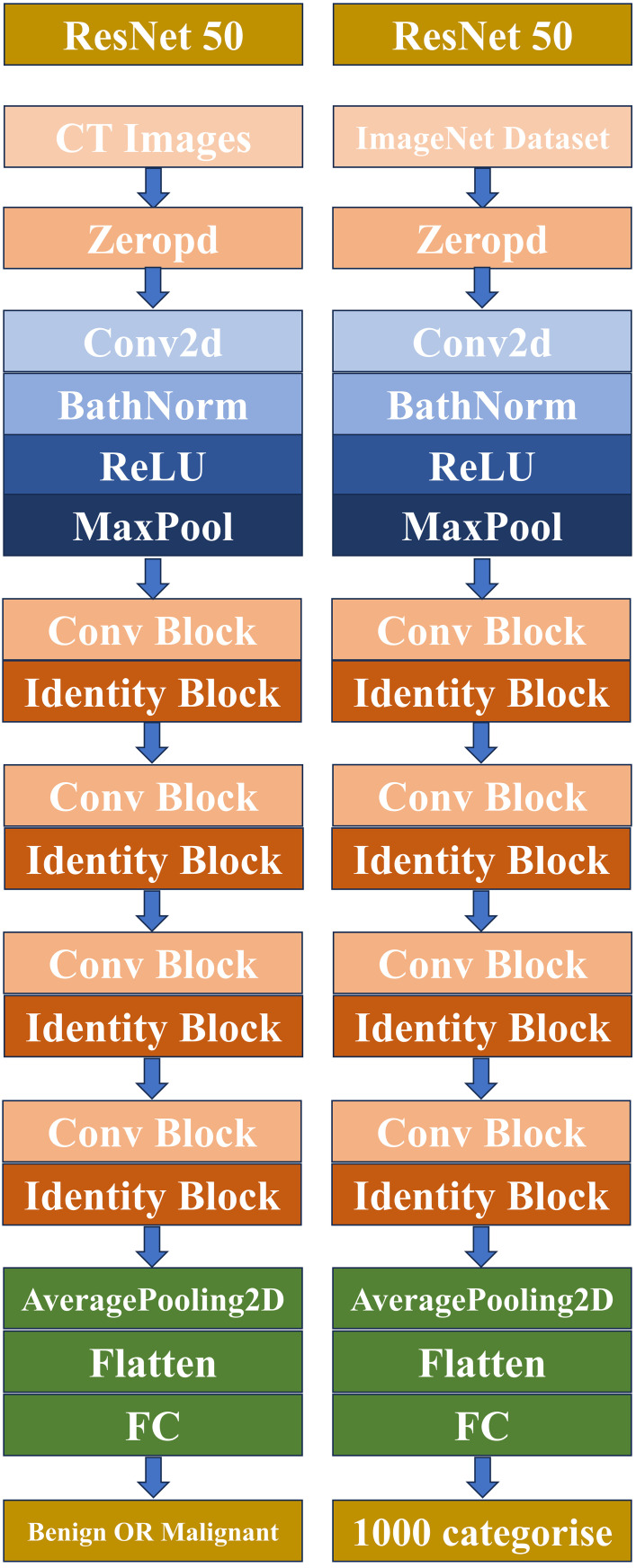
Schematic diagram of the deep convolutional neural network pretraining and fine-tuning network structure.

Before extracting deep learning (DL) features, the region of interest (ROI) with the largest sagittal plane area was selected for cropping. The input images were resampled to a size of 64×64 using linear interpolation, and the mean and standard deviation of pixel intensities were normalized to 0 and 1. The input images to the network were sagittal images, so the input channel was set to 1. Deep transfer learning (DTL) was applied using a method similar to early approaches, implemented in the PyTorch deep learning library (Python 3.6). ResNet50 (**Figures 4**, **5**) was selected as the base model for transfer learning. The model was trained using the Stochastic Gradient Descent (SGD) optimizer with an initial learning rate of 0.01, a batch size of 64, and a total of 50 epochs. The penultimate layer (average pooling layer) was selected as the transfer feature, and the model parameters were divided into two parts: (1) the backbone and (2) the task-specific part. The task-specific parameters were randomly initialized, and the backbone used the pre-trained parameters from the ImageNet model. Cosine annealing learning rate decay was used for the task-specific parameters.

### 2.7 Feature selection and fusion

To prevent data leakage, the entire dataset was first randomly divided into a training cohort (n=115) and a testing cohort (n=30). All subsequent feature selection steps were performed exclusively in the training cohort, and the testing cohort was used only for independent performance evaluation. To address class imbalance, the SMOTE algorithm was applied to the training cohort to balance the number of benign and malignant cases. The test cohort remained unchanged.

To ensure feature reproducibility and remove unstable features, intraclass correlation coefficients (ICCs) between HCR features were first calculated. HCR features with ICCs ≥ 0.8 were selected twice, reducing the number of features from 1834 to 724. To reduce redundancy among highly reproducible features, Spearman rank correlation coefficients were calculated to express relationships between features. Features with a correlation coefficient greater than 0.9 were filtered by retaining only one feature from each pair. To further eliminate redundant features while preserving representative ones in this high-dimensional setting, a greedy algorithm was used for feature selection, where the most redundant feature in the current feature set was removed. After filtering using the Spearman correlation coefficient and greedy selection, the number of features was reduced from 724 to 202. Finally, the Least Absolute Shrinkage and Selection Operator (LASSO) was employed for further dimensionality reduction and feature selection, as it can shrink irrelevant coefficients to zero and is well−suited for small−sample, high−dimensional data.the LASSO was used to shrink some regression coefficients to zero by constructing a penalty function (λ), thus including stable HCR features into LASSO-Cox analysis. The optimal λ value was determined using ten-fold cross-validation based on the minimum standard. The non-zero coefficients and their corresponding weights were selected.

### 2.8 Model construction and validation

After feature fusion and selection, several classification models were constructed using the scikit-learn machine learning library. The machine learning classification models included Support Vector Machine (SVM), K-Nearest Neighbors (KNN), Extremely Randomized Trees (ExtraTrees), and Multilayer Perceptron (MLP). All models were trained in the training set using a grid search algorithm and hyperparameter tuning for each model. To prevent overfitting, 5-fold cross-validation was performed to select the optimal parameters for each classification model. The optimal radiomics feature importance score for the fused features was obtained. Receiver Operating Characteristic (ROC) curves were plotted, and the Area Under the Curve (AUC), accuracy, sensitivity, and specificity were calculated to comprehensively evaluate the performance of each predictive model. A nomogram (DLRN) was created to visualize classification results by combining clinical baseline features with the fused feature model.

### 2.9 Statistical analysis

Univariate comparisons (using t-tests, the Mann–Whitney U test, and/or the chi-square test, depending on the case) were conducted to assess the differences in clinical characteristics and radiomics scores between malignant and benign tumors. To assess the diagnostic performance of both the radiomics model and the readers, ROC curves were generated. The AUC, accuracy, sensitivity, specificity, positive predictive value (PPV), and negative predictive value (NPV) were calculated for all three groups. The DeLong test was applied to compare the AUC values of the various models. The Hosmer–Lemeshow test was used to evaluate the calibration of the radiomics nomogram. Decision Curve Analysis (DCA) was performed to measure the net benefit of the models at varying threshold probabilities. All statistical analyses were performed using R software (version 3.5.2; www.r-project.org), with statistical significance set at *p* < 0.05.

## 3 Results

### 3.1 Clinical baseline characteristics

Among the 176 patients who met the inclusion criteria, 31 patients were excluded due to the following reasons: (1) the maximum lesion diameter was less than 0.5 cm (12 patients), and (2) poor image quality or presence of foreign object artifacts (19 patients). Ultimately, 145 patients were included in the study, consisting of 69 females and 76 males, with an average age of 43.64 ± 22.59 years. The average maximum tumor diameter was 2.74 ± 1.22 cm, and the average minimum diameter was 1.67 ± 0.74 cm. **Table 1** summarizes the baseline characteristics of benign and malignant orbital tumors in the training and test cohorts. Multivariate analysis following univariate screening (**Table 2**) confirmed that both homogeneous enhancement and ill-defined/infiltrative were independent CT features for differentiating benign from malignant tumors. Therefore, these two significant semantic features were selected for integration into the nomogram model. Although a high maximum tumor diameter is a well-established imaging biomarker associated with malignancy, as it reflects the rate of tumor progression (17), it was not retained as an independent clinical factor in our final multivariate model. Consequently, the maximum tumor diameter was not included as a separate component in the nomogram.

**Table 1:** Baseline patient characteristics in the training cohort and test cohort.

Characteristic	Training cohort			Test cohort		
	Benign(n=42)	Malignant(n=74)	*P*-value	Benign(n=10)	Malignant(n=19)	*P*-value
Age^#^	42.56 ± 17.88	42.91 ± 26.12	0.51	37.26 ± 19.98	52.21 ± 16.86	0.04
Maximum_diameter^#^	2.37 ± 0.85	2.85 ± 1.35	0.13	2.67 ± 1.08	3.18 ± 1.31	0.33
Minimum_diameter^#^	1.48 ± 0.55	1.76 ± 0.85	0.13	1.52 ± 0.40	1.79 ± 0.68	0.25
Gender			0.21			0.44
Female	24 (57.14%)	32 (43.24%)		3 (30.00%)	10 (52.63%)	
Male	18 (42.86%)	42 (56.76%)		7 (70.00%)	9 (47.37%)	
Extraconal	11 (26.19%)	20 (27.03%)	1	1 (10.00%)	3 (15.79%)	1
Intraconal	19 (45.24%)	21 (28.38%)	0.10	5 (50.00%)	6 (31.58%)	0.57
Trans-spatial	12 (28.57%)	33 (44.59%)	0.13	4 (40.00%)	10 (52.63%)	0.80
Ill-defined/infiltrative	13 (30.95%)	49 (66.22%)	<0.001	4 (40.00%)	13 (68.42%)	0.28
Well-defined	6 (14.29%)	23 (31.08%)	0.07	2 (20.00%)	5 (26.32%)	1
Heterogeneous	13 (30.95%)	24 (32.43%)	1	6 (60.00%)	5 (26.32%)	0.17
Calcification	9 (21.43%)	16 (21.62%)	1	2 (20.00%)	1 (5.26%)	0.55
Necrosis/cystic change	4 (9.52%)	10 (13.51%)	0.74	1 (10.00%)	2 (10.53%)	1
Mild enhancement	28 (66.67%)	52 (70.27%)	0.85	8 (80.00%)	11 (57.89%)	0.44
Significantly enhancement	11 (26.19%)	18 (24.32%)	1	1 (10.00%)	8 (42.11%)	0.18
Homogeneous enhancement	13 (30.95%)	43 (58.11%)	0.008	2 (20.00%)	12 (63.16%)	0.07

#Data are mean ± standard deviation (range).

**Table 2:** Clinically significant factors and independent predictors.

Characteristic	Univariate logistic regression		Multivariate logistic regression	
	OR (95% *CI*)	*p*_value	OR (95% *CI*)	*p*_value
age	1.010 (1.004-1.017)	0.009	0.993 (0.978-1.007)	0.4
gender	2.333 (1.468-3.710)	0.003	1.363 (0.636-2.921)	0.504
Maximum diameter	1.270 (1.131-1.426)	0.001	0.708 (0.409-1.225)	0.3
Minimum diameter	1.462 (1.213-1.761)	0.001	1.631 (0.678-3.927)	0.359
Significantly enhancement	1.636 (0.872-3.071)	0.198		
Heterogeneous enhancement	1.737 (1.081-2.790)	0.055		
Calcification	1.778 (0.896-3.529)	0.167		
Heterogeneous	1.846 (1.048-3.254)	0.075		
Necrosis/cystic change	2.500 (0.945-6.613)	0.121		
Extraconal	1.818 (0.980-3.370)	0.111		
Intraconal	1.105 (0.656-1.861)	0.752		
Trans-spatial	2.750 (1.579-4.787)	0.003	1.052 (0.421-2.63)	0.928
Mild enhancement	1.857 (1.262-2.732)	0.008	0.533 (0.245-1.163)	0.185
Homogeneous enhancement	3.308 (1.966-5.568)	0.000	4.001 (1.788-8.953)	**0.005**
Ill-defined/infiltrative	3.769 (2.257-6.297)	0.000	3.182 (1.293-7.83)	**0.034**
Well-defined	3.833 (1.804-8.150)	0.003	2.259 (0.713-7.156)	0.245

OR, Odds Ratio; CI, Confidence Interval. Variables with *P* < 0.05 in univariate analysis were included in the multivariate model. Bold values represent P<0.05 in the multivariate logistic regression.

### 3.2 Evaluation of conventional CT semantic features

Multivariate logistic regression analysis identified homogeneous enhancement and ill-defined/infiltrative as independent predictors for differentiating malignant from benign orbital tumors (**Table 2**). Specifically, lesions with homogeneous enhancement had an odds ratio (OR) of 4.001 (95% CI: 1.788-8.953; *P* = 0.005) for malignancy, while those with an ill-defined/infiltrative had an OR of 3.182 (95% CI: 1.293-7.83;*P* = 0.034).Based on these results, homogeneous enhancement and ill-defined/infiltrative—the features retained as independent in the multivariate model—were selected for integration into the subsequent clinical-semantic model and the final nomogram.

### 3.3 Feature selection

**Figure 6:**
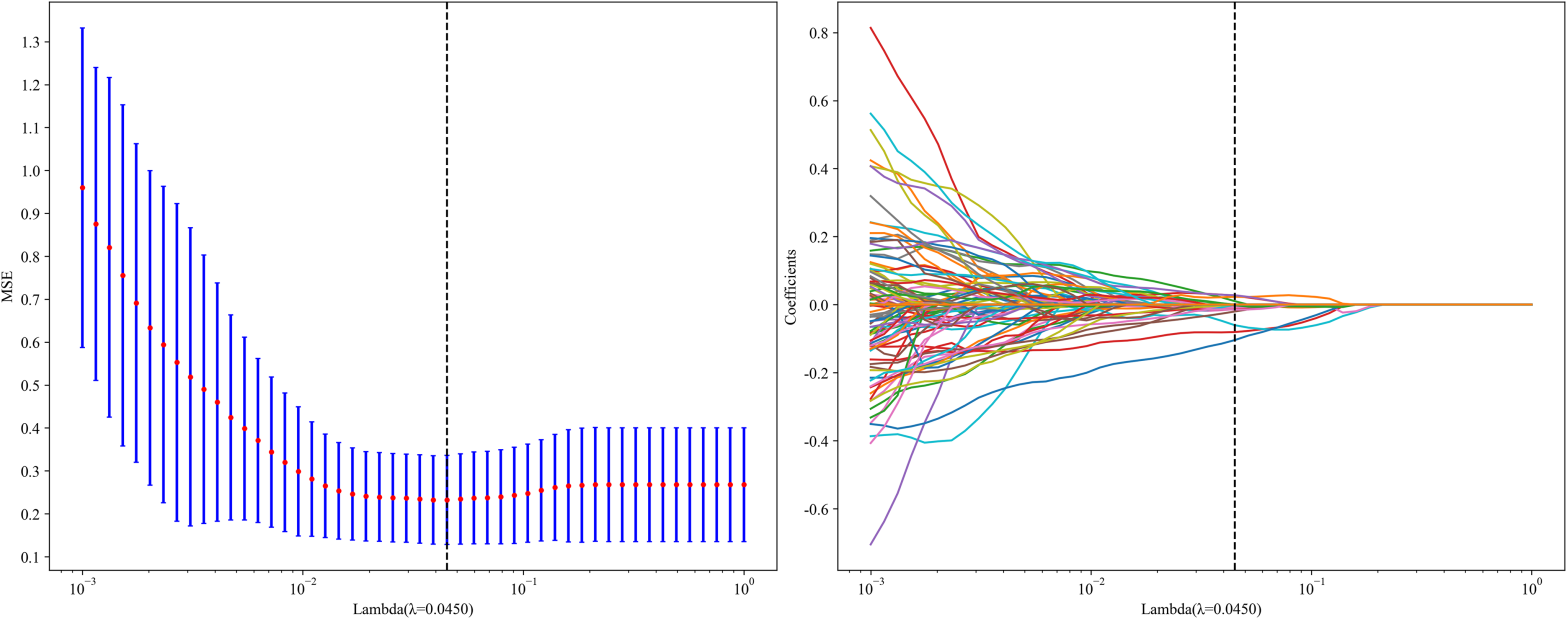
Hand-crafted radiomics feature selection using the least absolute shrinkage and the histogram of the Radiomics feature importance score based on the selected features. The optimal λ value of 0.0450 was selected.

LASSO regression analysis was used for dimensionality reduction of the 1834 HCR features. The penalty coefficient (λ = 0.0450) selection, feature selection process, and feature coefficient change curves with respect to λ are shown in **Figure 6**. After feature selection, a total of 14 HCR features were selected, including 3 first-order statistical features, 2 shape features, 2 GLDM features, 2 NGTDM features, 3 GLSZM features, and 2 GLRLM features. Radiomics feature importance scores were constructed using the 13 selected HCR features and their corresponding regression coefficients.

**Figure 7:**
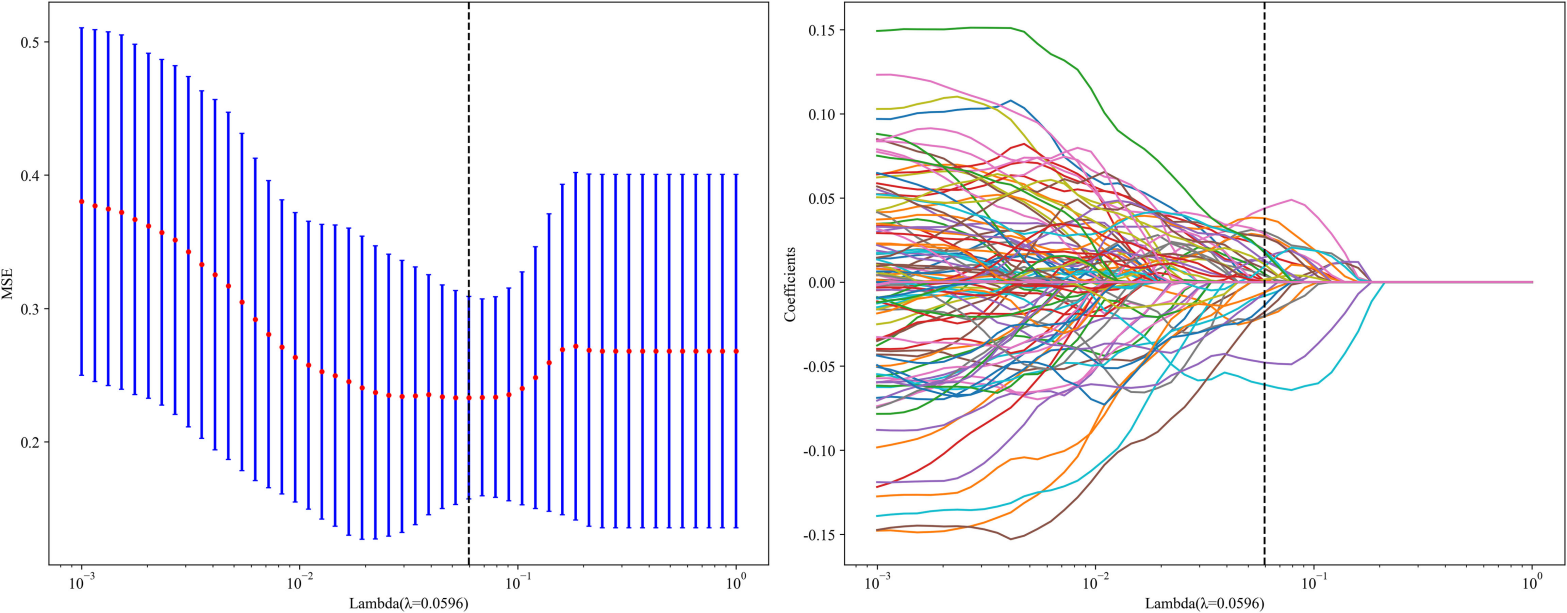
DL feature selection using the least absolute shrinkage and the histogram of the Deep Learning feature importance score based on the selected features. The optimal λvalue of 0.0596 was selected.

LASSO regression analysis was also applied to reduce the dimensionality of the 2048 DTL features. The penalty coefficient (λ = 0.0596) selection, feature selection process, and feature coefficient change curves with respect to λ are shown in **Figure 7**. After selection, 30 DTL features were chosen. The deep learning feature importance scores were constructed using the 30 DTL features and their corresponding regression coefficients.

**Figure 8:**
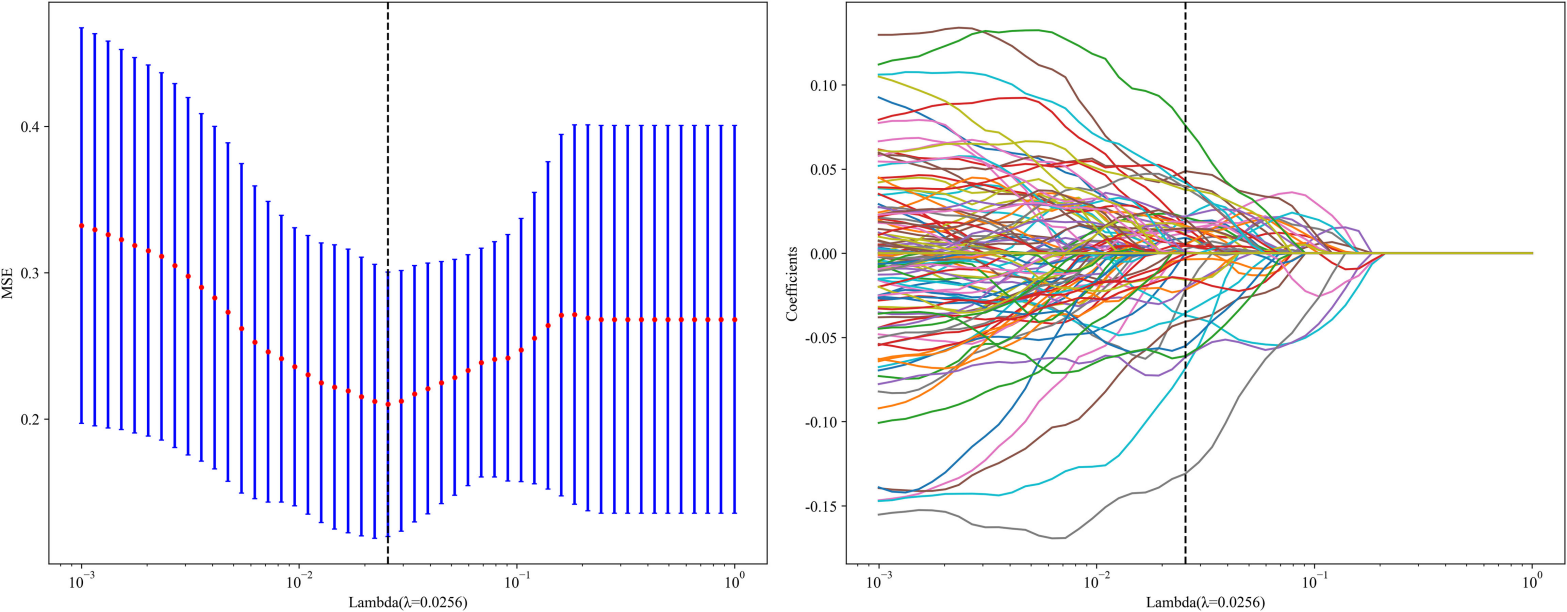
Fusion feature selection using the least absolute shrinkage and the histogram of the Deep Learning Radiomics feature importance score based on the selected features. The optimal λ value of 0.0256 was selected.

LASSO regression was used for dimensionality reduction of the 3882 fusion features (1,834 HCR features plus 2,048 DTL features). The penalty coefficient (λ = 0.0256) selection, feature selection process, and feature coefficient change curves with respect to λ are shown in **Figure 8**. After feature fusion and selection, 7 HCR features and 29 DTL features were retained. The fused features and their corresponding regression coefficients were used to construct the deep learning radiomics feature importance scores.

### 3.4 Model predictive performance

**Figure 9:**
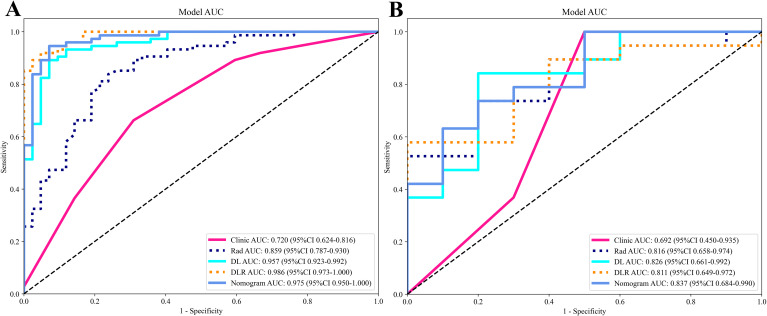
The AUCs of various prediction models in the training **(A)** and test **(B)** cohorts.

**Figure 10:**
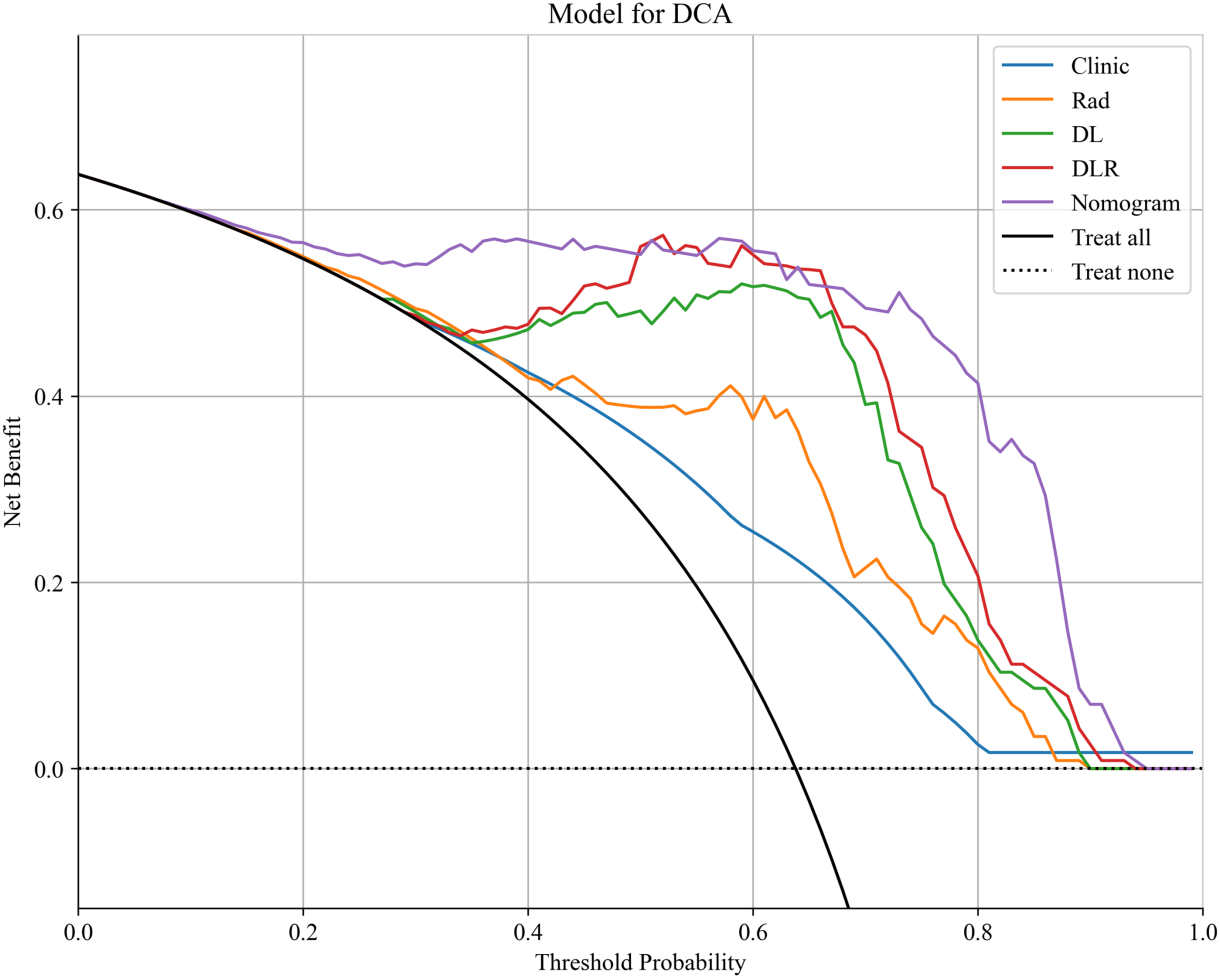
Decision curve analysis was developed in the training cohort with various prediction models.

**Figure 11:**
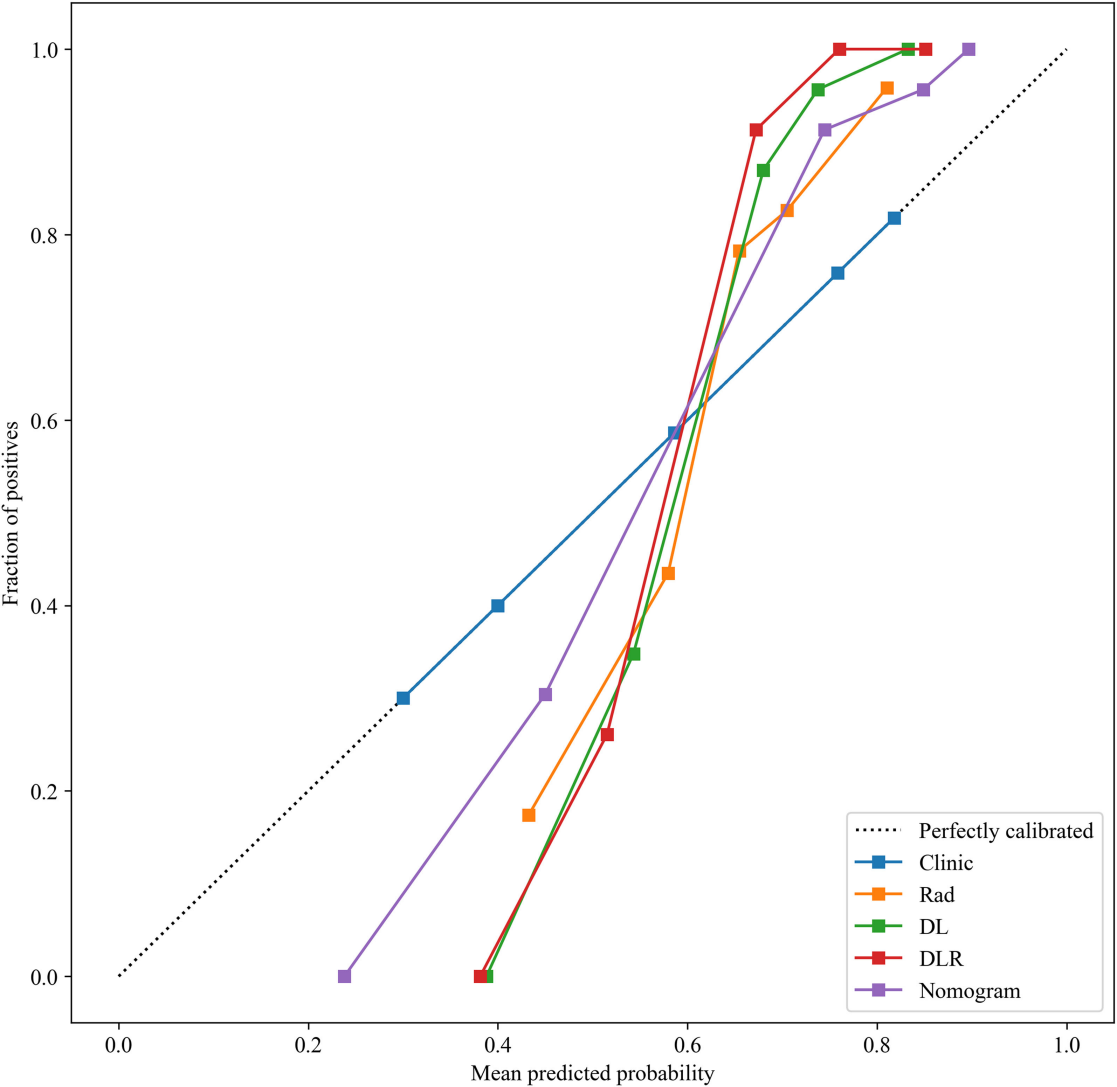
The calibration curve of the radiomics nomogram for the test cohort.

Based on the comparative analysis of machine learning algorithms presented in **Table 3**, the ExtraTrees classifier was selected for all subsequent modeling. This decision was based on its optimal balance between discriminative performance in the test cohort and generalization stability, as evidenced by having the smallest discrepancy in AUC between the training and test cohorts (ΔAUC = 0.043) among the evaluated models. While not achieving the absolute highest test AUC, this minimal performance gap indicates a substantially lower risk of overfitting, making ExtraTrees the most suitable and robust algorithm for building the final predictive models in this study. As shown in **Table 4**, **Figure 9**, the optimal prediction model was the feature fusion model, with an AUC of 0.986(95%CI,0.9729-1.0000) in the training cohort and 0.811(95%CI,0.6495-0.9716) in the test cohort. When the feature fusion model was combined with clinical data and significant CT semantic features, the nomogram produced an AUC of 0.975(95%CI,0.9496-1.0000) in the training cohort and 0.837(95%CI,0.6839-0.9898) in the test cohort. Using the DeLong test, there were no statistically significant differences in predictive performance between the feature fusion model and the nomogram in both the training and test cohorts (*P* values of 0.090 and 0.198, respectively). In contrast, differences between the other models in both cohorts were statistically significant (*P* < 0.05). Decision curve analysis (DCA) demonstrated that the nomogram was more beneficial to patients compared to the DL, traditional radiomics, and feature fusion prediction models (**Figure 10**). The calibration curve of the radiomics nomogram for the test cohort was plotted to assess the agreement between the predicted and actual outcomes. As shown in **Figure 11**, the calibration curve demonstrates the reliability of the nomogram in predicting the probability of malignancy in orbital tumors. A closer alignment of the curve with the diagonal line indicates better calibration, meaning the predicted probabilities are closer to the actual observed outcomes in the test cohort.

**Table 3:** Performance comparison of the four machine learning models in the test cohort.

Model	AUC(95%CI)	Accuracy	Sensitivity	Specificity	PPV	NPV
Training						
SVM	0.880 (0.7974-0.9626)	0.888	0.932	0.932	0.932	0.932
KNN	0.873 (0.8121-0.9334)	0.776	0.810	0.810	0.810	0.810
ExtraTrees	0.859 (0.7873-0.9302)	0.810	0.896	0.896	0.896	0.896
MLP	0.888 (0.8262-0.9498)	0.871	0.872	0.872	0.872	0.872
Test						
SVM	0.768 (0.5375-0.9993)	0.828	0.895	0.700	0.850	0.778
KNN	0.676 (0.4594-0.8932)	0.690	0.789	0.500	0.750	0.556
ExtraTrees	0.816 (0.6578-0.9738)	0.759	0.737	0.800	0.875	0.615
MLP	0.774 (0.5745-0.9728)	0.793	0.789	0.800	0.882	0.667

Data in parentheses are 95% confidence intervals.

DL, deep learning model; DLR, deep learning and Radiomics fused model.

AUC, area under the curve; CI, confidence interval; PPV, positive predictive value; NPV, negative predictive value.

**Table 4:** Diagnostic efficiency of different models in the training cohort and test cohort.

Model	AUC(95%CI)	Accuracy	Sensitivity	Specificity	PPV	NPV
Training						
Clinic	0.720 (0.6241-0.8161)	0.672	0.662	0.690	0.790	0.537
Radiomics	0.859 (0.7873-0.9302)	0.810	0.838	0.762	0.861	0.727
DL	0.957 (0.9228-0.9917)	0.905	0.892	0.929	0.957	0.830
DLR	0.986 (0.9729-1.0000)	0.931	0.919	0.952	0.971	0.870
Nomogram	0.975 (0.9496-1.0000)	0.940	0.946	0.929	0.959	0.907
Test						
Clinic	0.692 (0.4495-0.9347)	0.828	1.000	0.500	0.792	1.000
Radiomics	0.816 (0.6578-0.9738)	0.759	0.737	0.800	0.875	0.615
DL	0.826 (0.6606-0.9920)	0.828	0.842	0.800	0.889	0.727
DLR	0.811 (0.6495-0.9716)	0.724	0.579	1.000	1.000	0.556
Nomogram	0.837 (0.6839-0.9898)	0.759	0.737	0.800	0.875	0.615

Data in parentheses are 95% confidence intervals.

DL, deep learning model; DLR, deep learning and Radiomics fused model.

AUC, area under the curve; CI, confidence interval; PPV, positive predictive value; NPV, negative predictive value.

### 3.5 Deep learning radiomics nomogram construction

**Figure 12:**
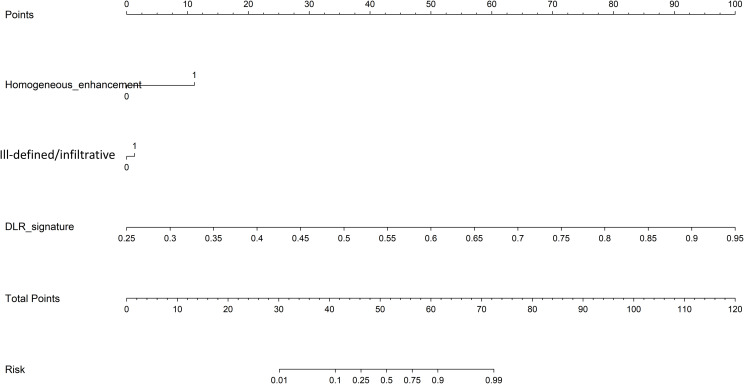
Deep learning radiomic nomogram was developed with the maximun diameter and feature fusion.

A comparison of the predictive performance of three models for benign and malignant orbital tumors showed that the feature fusion prediction model had the best performance and greater clinical utility. The nomogram constructed by combining feature fusion with clinical baseline characteristics can be used for visual distinction between malignant and benign orbital tumors (**Figure 12**).

## 4 Discussion

This study aimed to evaluate the application potential of deep learning radiomics (DL) and hand-crafted radiomics (HCR) features in distinguishing benign and malignant orbital tumors and to improve model predictive performance through feature fusion. We conducted a comprehensive performance comparison by constructing several machine learning classification models, including Support Vector Machine (SVM), K-Nearest Neighbors (KNN), Extremely Randomized Trees (ExtraTrees), and Multilayer Perceptron (MLP). The experimental results showed that the feature fusion model outperformed individual radiomics methods, and the nomogram (DLRN) built with clinical baseline features demonstrated high predictive value for practical clinical application.

Firstly, the feature fusion method showed good predictive performance with AUCs of 0.986(95%CI,0.9729-1.0000) in the training cohort and 0.811(95%CI,0.6495-0.9716) in the test cohort. Notably, when feature fusion was combined with clinical baseline data, the nomogram’s AUC further improved, achieving 0.975(95%CI,0.9496-1.0000) in the training cohort and 0.837(95%CI,0.6839-0.9898) in the test cohort. This result suggests that combining deep learning features with traditional radiomics features not only improves model diagnostic accuracy but also enhances predictive power by leveraging clinical data. It is noteworthy that a performance gap was observed for the fusion model between the training and test cohorts. This discrepancy may be attributed to several factors. First, although 145 patients were included, the sample size remains relatively limited given the high complexity of the deep learning model, which may have led to the learning of cohort-specific features and slightly reduced generalization on the independent test set. Second, orbital tumors exhibit inherent heterogeneity in pathology, growth patterns, and imaging manifestations, which could also affect the model’s stability in an external cohort. Crucially, the integration of the fusion model with clinical data into the nomogram not only improved the test AUC to 0.837 but also substantially narrowed this performance gap. This demonstrates that incorporating well-defined clinical and semantic features helps constrain model complexity and enhances both generalizability and clinical interpretability. Although previous orbital imaging studies have largely employed MRI or concentrated on limited tumor types, our work explores the use of routine contrast−enhanced CT combined with a clinically interpretable nomogram, representing a preliminary but potentially valuable addition to current knowledge.Future studies could further improve model robustness and external applicability by expanding multi-center sample sizes, adopting stricter cross-validation strategies, and exploring algorithms optimized for imbalanced data.

Among the different models evaluated, the ExtraTrees classifier demonstrated robust performance, particularly within the feature fusion and deep learning radiomics framework. ExtraTrees (Extremely Randomized Trees) is an ensemble learning method based on decision trees. Its core principle involves constructing a multitude of decision trees during training by introducing randomness in both the selection of data samples and the choice of split points for node splitting. For prediction, the outputs of all individual trees are aggregated, typically through majority voting for classification tasks. This ensemble strategy enhances the model’s generalization capability and stability (18).DeLong test results indicated that there was no statistically significant difference in predictive performance between the feature fusion model and the nomogram model in both the training and test sets (*P* values of 0.090 and 0.198, respectively), indicating comparable clinical value of the nomogram and feature fusion models in practical applications. Furthermore, the Hosmer−Lemeshow test showed non−significant results in both cohorts, and calibration curves confirmed good agreement between predicted probabilities and actual outcomes, indicating satisfactory model calibration.Decision curve analysis (DCA) further confirmed the higher net clinical benefit of the nomogram, highlighting its strong clinical potential, especially for patients who cannot undergo invasive procedures. Although its application in various ocular diseases has already demonstrated its value, research on orbital lesion analysis remains relatively scarce (19). The few published studies involve small patient sample sizes, focusing mainly on specific orbital sites or subtypes of tumors, such as lacrimal gland tumors, or concentrating solely on technical details (20–26).

Moreover, this study revealed that the fusion of deep transfer learning (DTL) and traditional radiomics features can effectively improve the ability to distinguish between benign and malignant orbital tumors. Convolutional Neural Networks (CNN), a type of deep learning model, are widely used in computer imaging and vision fields. In clinical practice, due to the difficulty in obtaining sufficiently large datasets, CNNs can only correctly learn features when trained with a large dataset. In preliminary studies, various CNN models, such as ResNet, VGGNet, etc., were tested. Compared with other CNN models, ResNet’s architecture employs shortcut connections, which facilitates effective feature fusion training. Additionally, the classic ResNet network structure includes ResNet-18, ResNet-34, ResNet-50, ResNet-101, and ResNet-152. ResNet-18 and ResNet-34 share the same basic structure and belong to relatively shallow networks. The latter three have different basic structures and are deeper networks. Since ResNet-101 and ResNet-152 have deeper network structures, they have more model parameters, making it more difficult to train with large datasets and reducing their performance. Therefore, ResNet-50, which is widely recognized, was ultimately chosen (27). Furthermore, deep transfer learning significantly improved feature extraction efficiency and accuracy by using pre-trained models (ResNet50) and fine-tuning them. Traditional radiomics methods, by extracting morphological features, gray-level co-occurrence matrices, etc., complement deep learning models in handling complex lesion shapes. The combination of both methods via feature fusion allows their advantages to complement each other, improving overall classification performance (28). Nowadays, deep learning radiomics methods are primarily used in tumor classification and prognostic research (29–31). In our study, we found that compared to using radiomics alone, the feature fusion model’s diagnostic performance was enhanced. Finally, we discovered that DLR has high predictive value for benign and malignant orbital tumor lesions, with AUCs of 0.981 and 0.761 in the training and test cohorts, respectively. Although the AUC between the nomogram and feature fusion models was not significantly different in both the training and test cohorts, DCA showed that the nomogram provided more benefits to patients. Moreover, the HCR and DTL feature extraction from routine contrast-enhanced CT images does not require special training, which presents substantial potential. To further improve model interpretability, we applied Grad−CAM to generate saliency heatmaps, which visually highlight the tumor regions most influential to the deep learning model’s decisions. This provides a more intuitive explanation of the abstract features extracted by deep transfer learning. While the interpretability of deep transfer learning features requires further investigation, it cannot be ruled out that the convolution operations in deep learning can map lesion characteristics, which could be used for constructing and classifying predictive models in the future.

test Although this study achieved favorable predictive results in most cases, several limitations should be acknowledged. First, this was a retrospective single-center study with a relatively small sample size (especially the test cohort) and imbalanced class distribution (97 malignant vs. 48 benign cases), which may limit generalizability and pose a risk of overfitting. Second, the current study focused mainly on CT imaging data and did not include other imaging modalities (e.g., MRI or ultrasound). Third, only the largest sagittal slice was used for deep transfer learning feature extraction rather than full volumetric information, which may have resulted in loss of spatial heterogeneity. Fourth, although we enhanced model transparency to some extent through feature importance ranking, integration with interpretable semantic and traditional radiomics features, and nomogram visualization, we were still unable to provide more intuitive visual explanations (e.g., pixel−level saliency heatmaps) for the abstract features extracted by deep transfer learning. This limitation stems from the methodological challenges posed by the inherent architecture of the pre−trained feature extractor, which complicates the direct mapping and interpretation of features back to specific regions in the original images. Future research should expand the sample size, conduct multicenter external validation, adopt class−balancing strategies, explore volumetric deep learning approaches, and incorporate dedicated explainability analysis techniques to provide finer−grained insights into the model’s decision−making process.In conclusion, this study demonstrates that the feature fusion method combining deep learning with traditional radiomics can significantly enhance the accuracy of differentiating benign and malignant orbital tumors and holds important clinical value. The deep learning radiomics nomogram (DLRN) provides a powerful tool for clinical decision-making in orbital tumors, especially for patients who cannot undergo invasive procedures.

## Data Availability

The original contributions presented in the study are included in the article/supplementary material. Further inquiries can be directed to the corresponding author.
